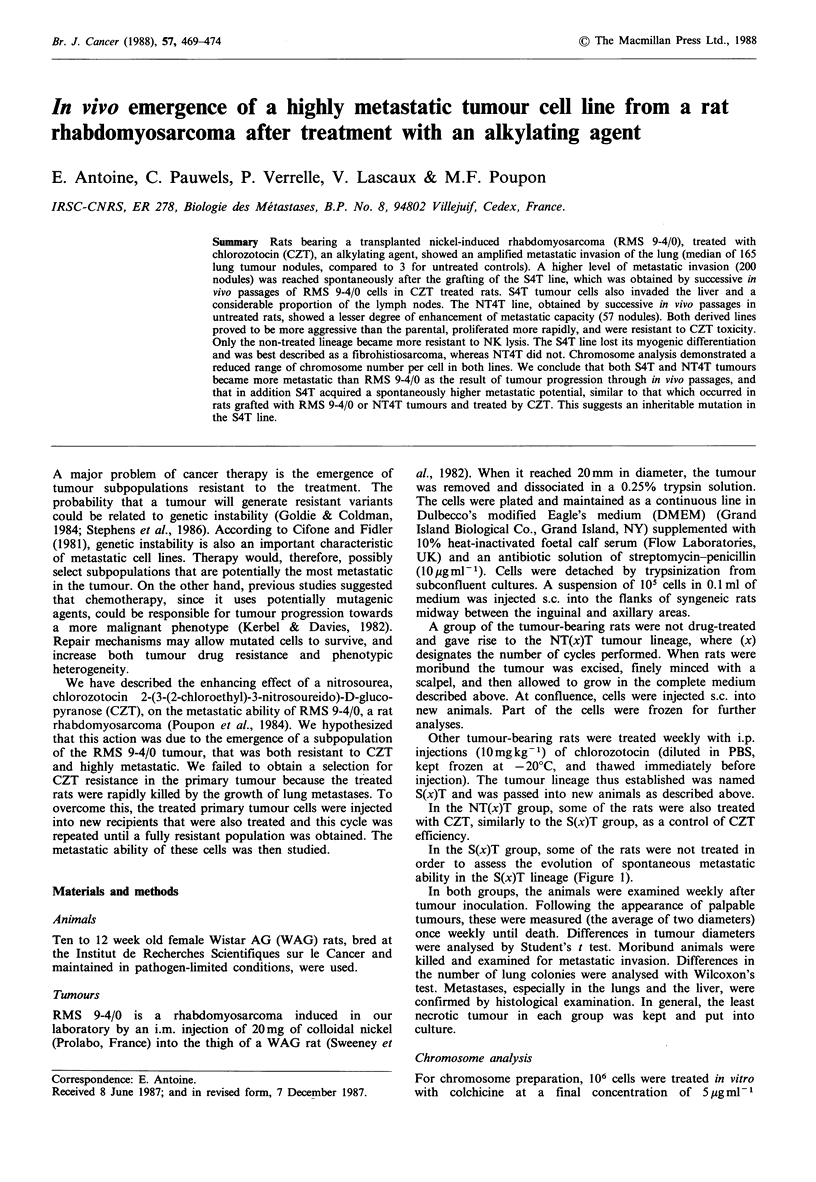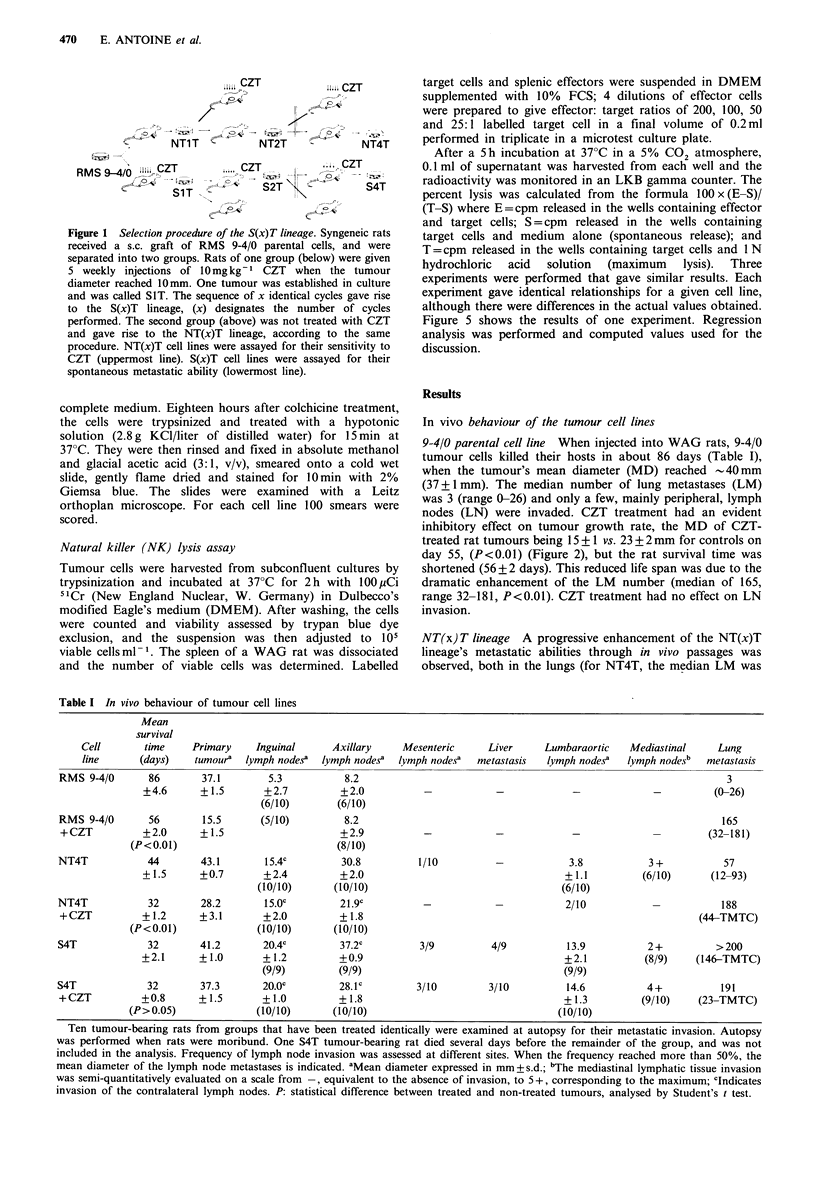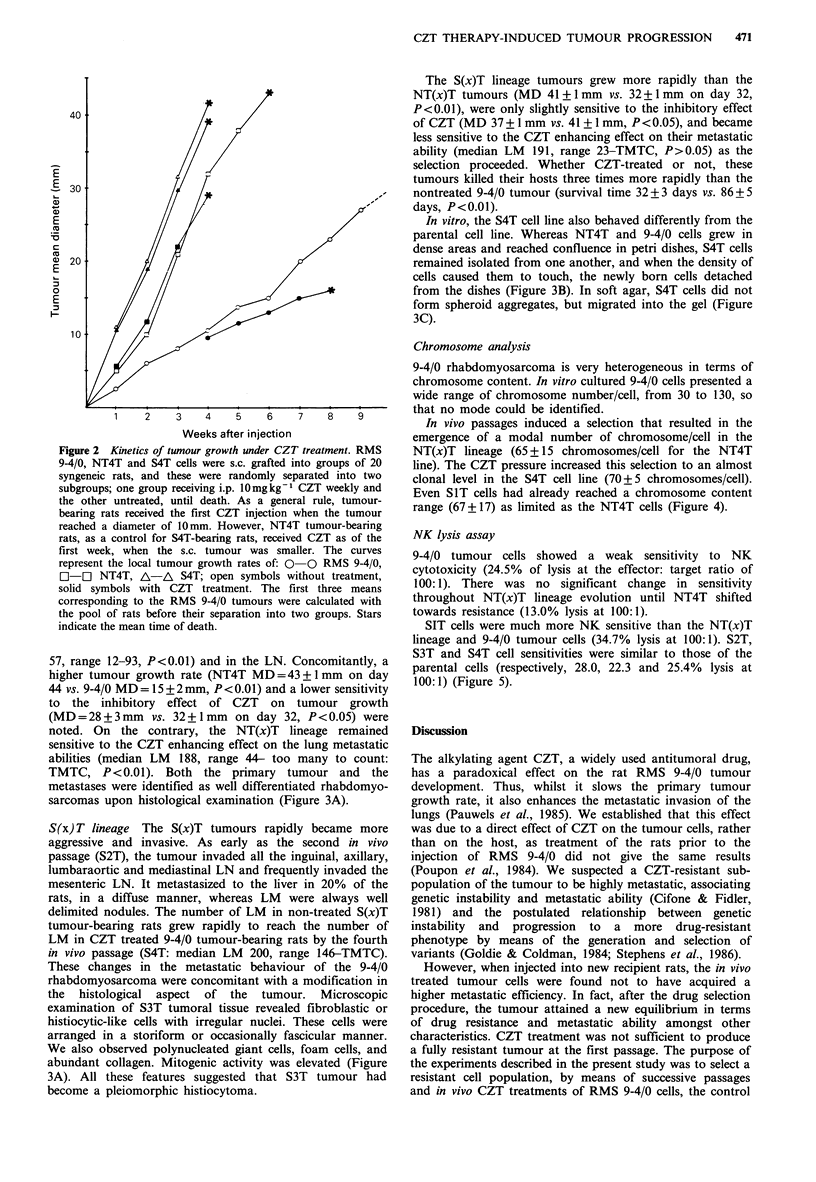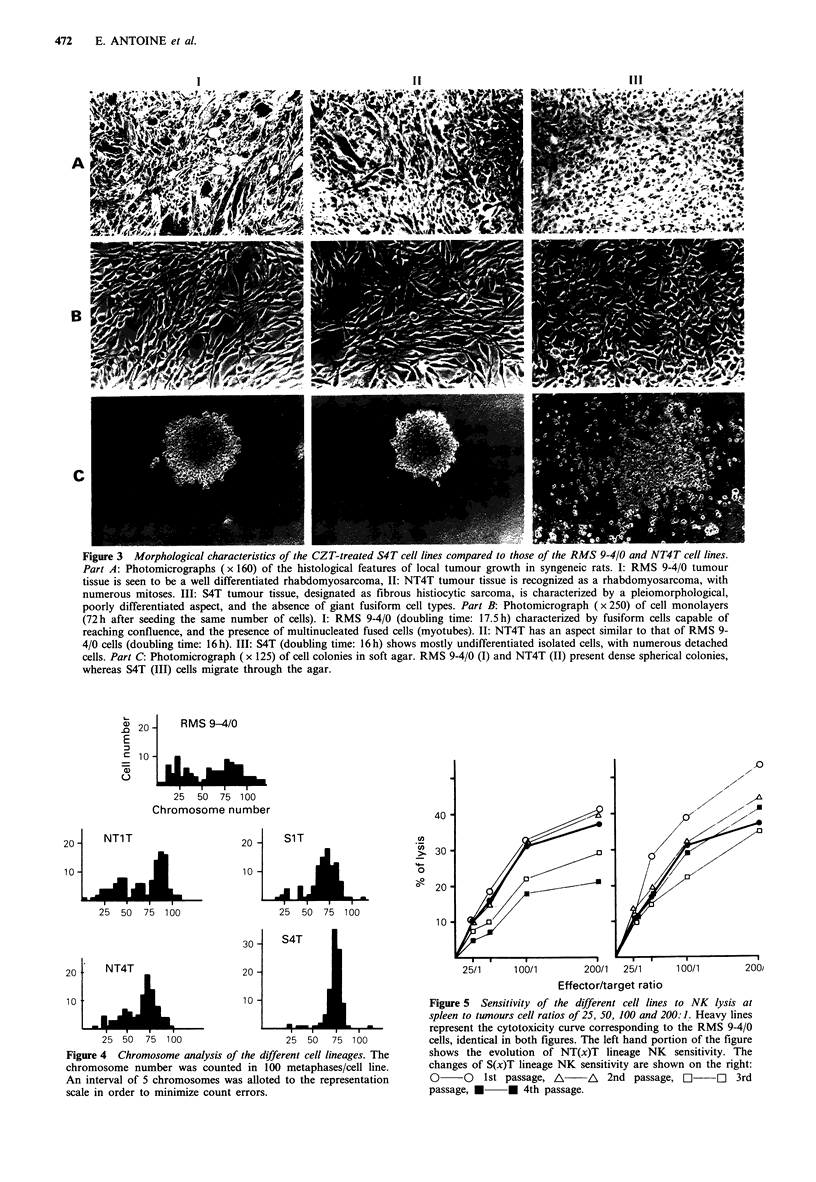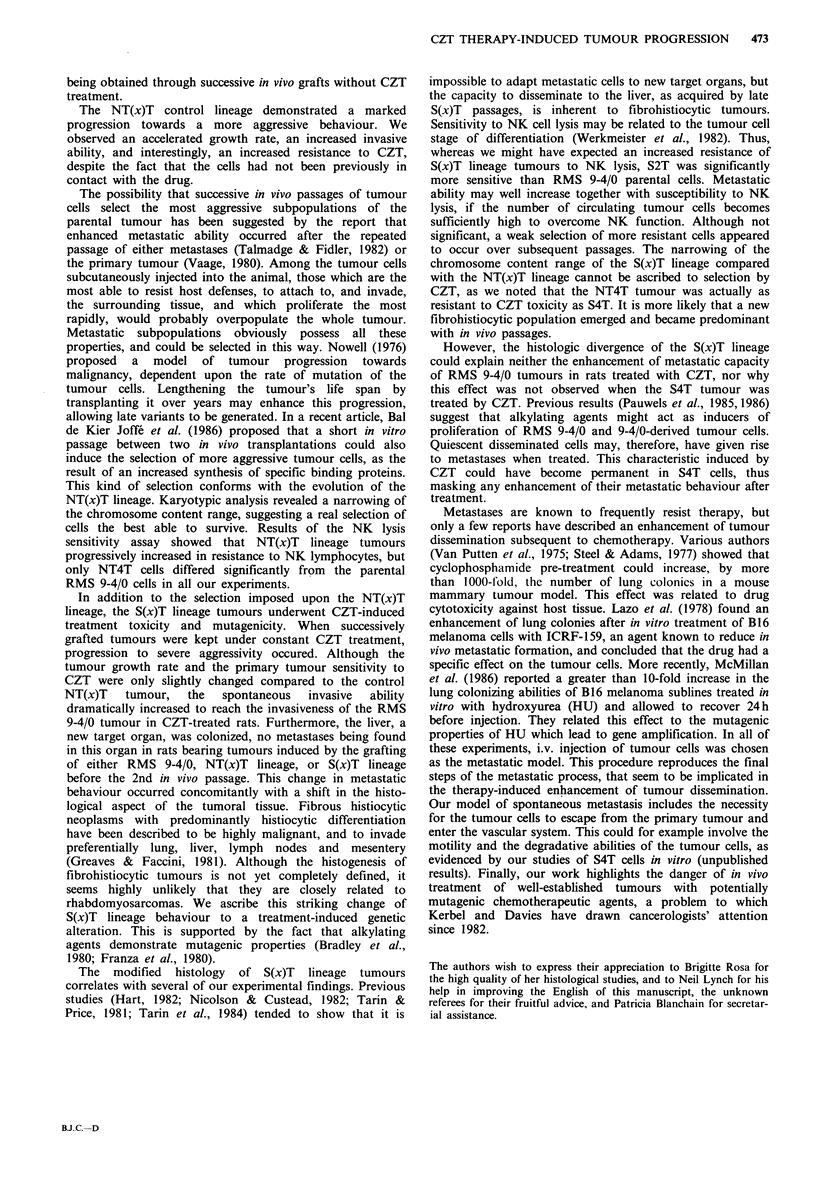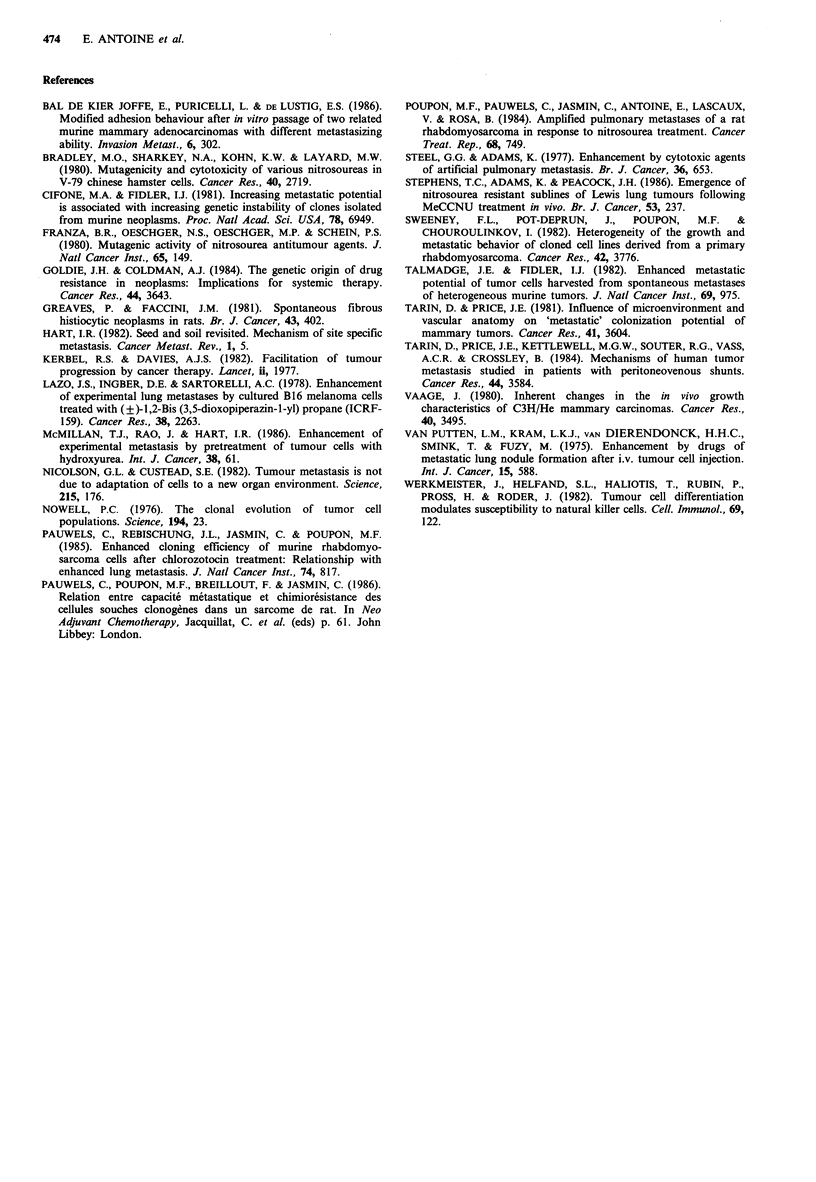# In vivo emergence of a highly metastatic tumour cell line from a rat rhabdomyosarcoma after treatment with an alkylating agent.

**DOI:** 10.1038/bjc.1988.109

**Published:** 1988-05

**Authors:** E. Antoine, C. Pauwels, P. Verrelle, V. Lascaux, M. F. Poupon

**Affiliations:** IRSC-CNRS, ER 278, Biologie des MÃ©tastases, Villejuif, France.

## Abstract

**Images:**


*Br. J. Cancer* (1988), **57**, 469–474 © The Macmillan Press Ltd., 1988

### *In vivo* emergence of a highly metastatic tumour cell line from a rat rhabdomyosarcoma after treatment with an alkylating agent

E. Antoine, C. Pauwels, P. Verrelle, V. Lascaux & M.F. Poupon

*IRSC-CNRS, ER 278, Biologie des Métastases, B.P. No. 8, 94802 Villejuif, Cedex, France.*

**Summary** Rats bearing a transplanted nickel-induced rhabdomyosarcoma (RMS 9-4/0), treated with chlorozotocin (CZT), an alkylating agent, showed an amplified metastatic invasion of the lung (median of 165 lung tumour nodules, compared to 3 for untreated controls). A higher level of metastatic invasion (200 nodules) was reached spontaneously after the grafting of the S4T line, which was obtained by successive *in vivo* passages of RMS 9-4/0 cells in CZT treated rats. S4T tumour cells also invaded the liver and a considerable proportion of the lymph nodes. The NT4T line, obtained by successive *in vivo* passages in untreated rats, showed a lesser degree of enhancement of metastatic capacity (57 nodules). Both derived lines proved to be more aggressive than the parental, proliferated more rapidly, and were resistant to CZT toxicity. Only the non-treated lineage became more resistant to NK lysis. The S4T line lost its myogenic differentiation and was best described as a fibrohistiosarcoma, whereas NT4T did not. Chromosome analysis demonstrated a reduced range of chromosome number per cell in both lines. We conclude that both S4T and NT4T tumours became more metastatic than RMS 9-4/0 as the result of tumour progression through *in vivo* passages, and that in addition S4T acquired a spontaneously higher metastatic potential, similar to that which occurred in rats grafted with RMS 9-4/0 or NT4T tumours and treated by CZT. This suggests an inheritable mutation in the S4T line.

A major problem of cancer therapy is the emergence of tumour subpopulations resistant to the treatment. The probability that a tumour will generate resistant variants could be related to genetic instability (Goldie & Coldman, 1984; Stephens *et al.*, 1986). According to Cifone and Fidler (1981), genetic instability is also an important characteristic of metastatic cell lines. Therapy would, therefore, possibly select subpopulations that are potentially the most metastatic in the tumour. On the other hand, previous studies suggested that chemotherapy, since it uses potentially mutagenic agents, could be responsible for tumour progression towards a more malignant phenotype (Kerbel & Davies, 1982). Repair mechanisms may allow mutated cells to survive, and increase both tumour drug resistance and phenotypic heterogeneity.

We have described the enhancing effect of a nitrosourea, chlorozotocin 2-(3-(2-chloroethyl)-3-nitrosoureido)-D-glucopyranose (CZT), on the metastatic ability of RMS 9-4/0, a rat rhabdomyosarcoma (Poupon *et al.*, 1984). We hypothesized that this action was due to the emergence of a subpopulation of the RMS 9-4/0 tumour, that was both resistant to CZT and highly metastatic. We failed to obtain a selection for CZT resistance in the primary tumour because the treated rats were rapidly killed by the growth of lung metastases. To overcome this, the treated primary tumour cells were injected into new recipients that were also treated and this cycle was repeated until a fully resistant population was obtained. The metastatic ability of these cells was then studied.

### Materials and methods

### *Animals*

Ten to 12 week old female Wistar AG (WAG) rats, bred at the Institut de Recherches Scientifiques sur le Cancer and maintained in pathogen-limited conditions, were used.

### *Tumours*

RMS 9-4/0 is a rhabdomyosarcoma induced in our laboratory by an i.m. injection of 20 mg of colloidal nickel (Prolabo, France) into the thigh of a WAG rat (Sweeney *et al.*, 1982). When it reached 20 mm in diameter, the tumour was removed and dissociated in a 0.25% trypsin solution. The cells were plated and maintained as a continuous line in Dulbecco's modified Eagle's medium (DMEM) (Grand Island Biological Co., Grand Island, NY) supplemented with 10% heat-inactivated foetal calf serum (Flow Laboratories, UK) and an antibiotic solution of streptomycin–penicillin (10 $\mu$g ml$^{-1}$). Cells were detached by trypsinization from subconfluent cultures. A suspension of $10^5$ cells in 0.1 ml of medium was injected s.c. into the flanks of syngeneic rats midway between the inguinal and axillary areas.

A group of the tumour-bearing rats were not drug-treated and gave rise to the NT($x$)T tumour lineage, where ($x$) designates the number of cycles performed. When rats were moribund the tumour was excised, finely minced with a scalpel, and then allowed to grow in the complete medium described above. At confluence, cells were injected s.c. into new animals. Part of the cells were frozen for further analyses.

Other tumour-bearing rats were treated weekly with i.p. injections (10 mg kg$^{-1}$) of chlorozotocin (diluted in PBS, kept frozen at $-20$°C, and thawed immediately before injection). The tumour lineage thus established was named S($x$)T and was passed into new animals as described above.

In the NT($x$)T group, some of the rats were also treated with CZT, similarly to the S($x$)T group, as a control of CZT efficiency.

In the S($x$)T group, some of the rats were not treated in order to assess the evolution of spontaneous metastatic ability in the S($x$)T lineage (Figure 1).

In both groups, the animals were examined weekly after tumour inoculation. Following the appearance of palpable tumours, these were measured (the average of two diameters) once weekly until death. Differences in tumour diameters were analysed by Student's $t$ test. Moribund animals were killed and examined for metastatic invasion. Differences in the number of lung colonies were analysed with Wilcoxon's test. Metastases, especially in the lungs and the liver, were confirmed by histological examination. In general, the least necrotic tumour in each group was kept and put into culture.

### *Chromosome analysis*

For chromosome preparation, $10^6$ cells were treated *in vitro* with colchicine at a final concentration of 5 $\mu$g ml$^{-1}$

Correspondence: E. Antoine.
Received 8 June 1987; and in revised form, 7 December 1987.

**Figure 1** *Selection procedure of the S(x)T lineage.* Syngeneic rats received a s.c. graft of RMS 9-4/0 parental cells, and were separated into two groups. Rats of one group (below) were given 5 weekly injections of $10\,mg\,kg^{-1}$ CZT when the tumour diameter reached 10 mm. One tumour was established in culture and was called S1T. The sequence of *x* identical cycles gave rise to the S(*x*)T lineage, (*x*) designates the number of cycles performed. The second group (above) was not treated with CZT and gave rise to the NT(*x*)T lineage, according to the same procedure. NT(*x*)T cell lines were assayed for their sensitivity to CZT (uppermost line). S(*x*)T cell lines were assayed for their spontaneous metastatic ability (lowermost line).

complete medium. Eighteen hours after colchicine treatment, the cells were trypsinized and treated with a hypotonic solution (2.8 g KCl/liter of distilled water) for 15 min at 37°C. They were then rinsed and fixed in absolute methanol and glacial acetic acid (3:1, v/v), smeared onto a cold wet slide, gently flame dried and stained for 10 min with 2% Giemsa blue. The slides were examined with a Leitz orthoplan microscope. For each cell line 100 smears were scored.

### *Natural killer (NK) lysis assay*

Tumour cells were harvested from subconfluent cultures by trypsinization and incubated at 37°C for 2 h with 100 $\mu$Ci $^{51}Cr$ (New England Nuclear, W. Germany) in Dulbecco's modified Eagle's medium (DMEM). After washing, the cells were counted and viability assessed by trypan blue dye exclusion, and the suspension was then adjusted to $10^5$ viable cells $ml^{-1}$. The spleen of a WAG rat was dissociated and the number of viable cells was determined. Labelled target cells and splenic effectors were suspended in DMEM supplemented with 10% FCS; 4 dilutions of effector cells were prepared to give effector: target ratios of 200, 100, 50 and 25:1 labelled target cell in a final volume of 0.2 ml performed in triplicate in a microtest culture plate.

After a 5 h incubation at 37°C in a 5% $CO_2$ atmosphere, 0.1 ml of supernatant was harvested from each well and the radioactivity was monitored in an LKB gamma counter. The percent lysis was calculated from the formula $100 \times (E-S)/(T-S)$ where E = cpm released in the wells containing effector and target cells; S = cpm released in the wells containing target cells and medium alone (spontaneous release); and T = cpm released in the wells containing target cells and 1 N hydrochloric acid solution (maximum lysis). Three experiments were performed that gave similar results. Each experiment gave identical relationships for a given cell line, although there were differences in the actual values obtained. Figure 5 shows the results of one experiment. Regression analysis was performed and computed values used for the discussion.

### Results

### In vivo *behaviour of the tumour cell lines*

*9-4/0 parental cell line* When injected into WAG rats, 9-4/0 tumour cells killed their hosts in about 86 days (Table I), when the tumour's mean diameter (MD) reached ~40 mm (37±1 mm). The median number of lung metastases (LM) was 3 (range 0–26) and only a few, mainly peripheral, lymph nodes (LN) were invaded. CZT treatment had an evident inhibitory effect on tumour growth rate, the MD of CZT-treated rat tumours being 15±1 *vs.* 23±2 mm for controls on day 55, ($P<0.01$) (Figure 2), but the rat survival time was shortened (56±2 days). This reduced life span was due to the dramatic enhancement of the LM number (median of 165, range 32–181, $P<0.01$). CZT treatment had no effect on LN invasion.

*NT(x)T lineage* A progressive enhancement of the NT(*x*)T lineage's metastatic abilities through *in vivo* passages was observed, both in the lungs (for NT4T, the median LM was

**Table I** *In vivo* behaviour of tumour cell lines

| *Cell line* | *Mean survival time (days)* | *Primary tumour*[a] | *Inguinal lymph nodes*[a] | *Axillary lymph nodes*[a] | *Mesenteric lymph nodes*[a] | *Liver metastasis* | *Lumbaraortic lymph nodes*[a] | *Mediastinal lymph nodes*[b] | *Lung metastasis* |
|---|---|---|---|---|---|---|---|---|---|
| RMS 9-4/0 | 86 ±4.6 | 37.1 ±1.5 | 5.3 ±2.7 (6/10) | 8.2 ±2.0 (6/10) | – | – | – | – | 3 (0–26) |
| RMS 9-4/0 +CZT | 56 ±2.0 ($P<0.01$) | 15.5 ±1.5 | (5/10) | 8.2 ±2.9 (8/10) | – | – | – | – | 165 (32–181) |
| NT4T | 44 ±1.5 | 43.1 ±0.7 | 15.4[c] ±2.4 (10/10) | 30.8 ±2.0 (10/10) | 1/10 | – | 3.8 ±1.1 (6/10) | 3+ (6/10) | 57 (12–93) |
| NT4T +CZT | 32 ±1.2 ($P<0.01$) | 28.2 ±3.1 | 15.0[c] ±2.0 (10/10) | 21.9[c] ±1.8 (10/10) | – | – | 2/10 | – | 188 (44–TMTC) |
| S4T | 32 ±2.1 | 41.2 ±1.0 | 20.4[c] ±1.2 (9/9) | 37.2[c] ±0.9 (9/9) | 3/9 | 4/9 | 13.9 ±2.1 (9/9) | 2+ (8/9) | >200 (146–TMTC) |
| S4T +CZT | 32 ±0.8 ($P>0.05$) | 37.3 ±1.5 | 20.0[c] ±1.0 (10/10) | 28.1[c] ±1.8 (10/10) | 3/10 | 3/10 | 14.6 ±1.3 (10/10) | 4+ (9/10) | 191 (23–TMTC) |

Ten tumour-bearing rats from groups that have been treated identically were examined at autopsy for their metastatic invasion. Autopsy was performed when rats were moribund. One S4T tumour-bearing rat died several days before the remainder of the group, and was not included in the analysis. Frequency of lymph node invasion was assessed at different sites. When the frequency reached more than 50%, the mean diameter of the lymph node metastases is indicated. [a]Mean diameter expressed in mm±s.d.; [b]The mediastinal lymphatic tissue invasion was semi-quantitatively evaluated on a scale from –, equivalent to the absence of invasion, to 5+, corresponding to the maximum; [c]Indicates invasion of the contralateral lymph nodes. *P*: statistical difference between treated and non-treated tumours, analysed by Student's *t* test.

**Figure 2** *Kinetics of tumour growth under CZT treatment.* RMS 9-4/0, NT4T and S4T cells were s.c. grafted into groups of 20 syngeneic rats, and these were randomly separated into two subgroups; one group receiving i.p. $10\,mg\,kg^{-1}$ CZT weekly and the other untreated, until death. As a general rule, tumour-bearing rats received the first CZT injection when the tumour reached a diameter of 10 mm. However, NT4T tumour-bearing rats, as a control for S4T-bearing rats, received CZT as of the first week, when the s.c. tumour was smaller. The curves represent the local tumour growth rates of: ○—○ RMS 9-4/0, □—□ NT4T, △—△ S4T; open symbols without treatment, solid symbols with CZT treatment. The first three means corresponding to the RMS 9-4/0 tumours were calculated with the pool of rats before their separation into two groups. Stars indicate the mean time of death.

57, range 12–93, $P<0.01$) and in the LN. Concomitantly, a higher tumour growth rate (NT4T MD $=43\pm1$ mm on day 44 *vs.* 9-4/0 MD $=15\pm2$ mm, $P<0.01$) and a lower sensitivity to the inhibitory effect of CZT on tumour growth (MD $=28\pm3$ mm *vs.* $32\pm1$ mm on day 32, $P<0.05$) were noted. On the contrary, the NT(*x*)T lineage remained sensitive to the CZT enhancing effect on the lung metastatic abilities (median LM 188, range 44– too many to count: TMTC, $P<0.01$). Both the primary tumour and the metastases were identified as well differentiated rhabdomyosarcomas upon histological examination (Figure 3A).

*S(*x*)T lineage* The S(*x*)T tumours rapidly became more aggressive and invasive. As early as the second *in vivo* passage (S2T), the tumour invaded all the inguinal, axillary, lumbaraortic and mediastinal LN and frequently invaded the mesenteric LN. It metastasized to the liver in 20% of the rats, in a diffuse manner, whereas LM were always well delimited nodules. The number of LM in non-treated S(*x*)T tumour-bearing rats grew rapidly to reach the number of LM in CZT treated 9-4/0 tumour-bearing rats by the fourth *in vivo* passage (S4T: median LM 200, range 146–TMTC). These changes in the metastatic behaviour of the 9-4/0 rhabdomyosarcoma were concomitant with a modification in the histological aspect of the tumour. Microscopic examination of S3T tumoral tissue revealed fibroblastic or histiocytic-like cells with irregular nuclei. These cells were arranged in a storiform or occasionally fascicular manner. We also observed polynucleated giant cells, foam cells, and abundant collagen. Mitogenic activity was elevated (Figure 3A). All these features suggested that S3T tumour had become a pleiomorphic histiocytoma.

The S(*x*)T lineage tumours grew more rapidly than the NT(*x*)T tumours (MD $41\pm1$ mm *vs.* $32\pm1$ mm on day 32, $P<0.01$), were only slightly sensitive to the inhibitory effect of CZT (MD $37\pm1$ mm *vs.* $41\pm1$ mm, $P<0.05$), and became less sensitive to the CZT enhancing effect on their metastatic ability (median LM 191, range 23–TMTC, $P>0.05$) as the selection proceeded. Whether CZT-treated or not, these tumours killed their hosts three times more rapidly than the nontreated 9-4/0 tumour (survival time $32\pm3$ days *vs.* $86\pm5$ days, $P<0.01$).

*In vitro*, the S4T cell line also behaved differently from the parental cell line. Whereas NT4T and 9-4/0 cells grew in dense areas and reached confluence in petri dishes, S4T cells remained isolated from one another, and when the density of cells caused them to touch, the newly born cells detached from the dishes (Figure 3B). In soft agar, S4T cells did not form spheroid aggregates, but migrated into the gel (Figure 3C).

*Chromosome analysis*

9-4/0 rhabdomyosarcoma is very heterogeneous in terms of chromosome content. *In vitro* cultured 9-4/0 cells presented a wide range of chromosome number/cell, from 30 to 130, so that no mode could be identified.

*In vivo* passages induced a selection that resulted in the emergence of a modal number of chromosome/cell in the NT(*x*)T lineage ($65\pm15$ chromosomes/cell for the NT4T line). The CZT pressure increased this selection to an almost clonal level in the S4T cell line ($70\pm5$ chromosomes/cell). Even S1T cells had already reached a chromosome content range ($67\pm17$) as limited as the NT4T cells (Figure 4).

*NK lysis assay*

9-4/0 tumour cells showed a weak sensitivity to NK cytotoxicity (24.5% of lysis at the effector: target ratio of 100:1). There was no significant change in sensitivity throughout NT(*x*)T lineage evolution until NT4T shifted towards resistance (13.0% lysis at 100:1).

SIT cells were much more NK sensitive than the NT(*x*)T lineage and 9-4/0 tumour cells (34.7% lysis at 100:1). S2T, S3T and S4T cell sensitivities were similar to those of the parental cells (respectively, 28.0, 22.3 and 25.4% lysis at 100:1) (Figure 5).

### Discussion

The alkylating agent CZT, a widely used antitumoral drug, has a paradoxical effect on the rat RMS 9-4/0 tumour development. Thus, whilst it slows the primary tumour growth rate, it also enhances the metastatic invasion of the lungs (Pauwels *et al.*, 1985). We established that this effect was due to a direct effect of CZT on the tumour cells, rather than on the host, as treatment of the rats prior to the injection of RMS 9-4/0 did not give the same results (Poupon *et al.*, 1984). We suspected a CZT-resistant subpopulation of the tumour to be highly metastatic, associating genetic instability and metastatic ability (Cifone & Fidler, 1981) and the postulated relationship between genetic instability and progression to a more drug-resistant phenotype by means of the generation and selection of variants (Goldie & Coldman, 1984; Stephens *et al.*, 1986).

However, when injected into new recipient rats, the *in vivo* treated tumour cells were found not to have acquired a higher metastatic efficiency. In fact, after the drug selection procedure, the tumour attained a new equilibrium in terms of drug resistance and metastatic ability amongst other characteristics. CZT treatment was not sufficient to produce a fully resistant tumour at the first passage. The purpose of the experiments described in the present study was to select a resistant cell population, by means of successive passages and *in vivo* CZT treatments of RMS 9-4/0 cells, the control

**Figure 3** *Morphological characteristics of the CZT-treated S4T cell lines compared to those of the RMS 9-4/0 and NT4T cell lines. Part A*: Photomicrographs (× 160) of the histological features of local tumour growth in syngeneic rats. I: RMS 9-4/0 tumour tissue is seen to be a well differentiated rhabdomyosarcoma, II: NT4T tumour tissue is recognized as a rhabdomyosarcoma, with numerous mitoses. III: S4T tumour tissue, designated as fibrous histiocytic sarcoma, is characterized by a pleiomorphological, poorly differentiated aspect, and the absence of giant fusiform cell types. *Part B*: Photomicrograph (× 250) of cell monolayers (72 h after seeding the same number of cells). I: RMS 9-4/0 (doubling time: 17.5 h) characterized by fusiform cells capable of reaching confluence, and the presence of multinucleated fused cells (myotubes). II: NT4T has an aspect similar to that of RMS 9-4/0 cells (doubling time: 16 h). III: S4T (doubling time: 16 h) shows mostly undifferentiated isolated cells, with numerous detached cells. *Part C*: Photomicrograph (× 125) of cell colonies in soft agar. RMS 9-4/0 (I) and NT4T (II) present dense spherical colonies, whereas S4T (III) cells migrate through the agar.

**Figure 4** *Chromosome analysis of the different cell lineages.* The chromosome number was counted in 100 metaphases/cell line. An interval of 5 chromosomes was alloted to the representation scale in order to minimize count errors.

**Figure 5** *Sensitivity of the different cell lines to NK lysis at spleen to tumours cell ratios of 25, 50, 100 and 200:1.* Heavy lines represent the cytotoxicity curve corresponding to the RMS 9-4/0 cells, identical in both figures. The left hand portion of the figure shows the evolution of NT($x$)T lineage NK sensitivity. The changes of S($x$)T lineage NK sensitivity are shown on the right: ○——○ 1st passage, △——△ 2nd passage, □——□ 3rd passage, ■——■ 4th passage.

being obtained through successive *in vivo* grafts without CZT treatment.

The NT($x$)T control lineage demonstrated a marked progression towards a more aggressive behaviour. We observed an accelerated growth rate, an increased invasive ability, and interestingly, an increased resistance to CZT, despite the fact that the cells had not been previously in contact with the drug.

The possibility that successive *in vivo* passages of tumour cells select the most aggressive subpopulations of the parental tumour has been suggested by the report that enhanced metastatic ability occurred after the repeated passage of either metastases (Talmadge & Fidler, 1982) or the primary tumour (Vaage, 1980). Among the tumour cells subcutaneously injected into the animal, those which are the most able to resist host defenses, to attach to, and invade, the surrounding tissue, and which proliferate the most rapidly, would probably overpopulate the whole tumour. Metastatic subpopulations obviously possess all these properties, and could be selected in this way. Nowell (1976) proposed a model of tumour progression towards malignancy, dependent upon the rate of mutation of the tumour cells. Lengthening the tumour's life span by transplanting it over years may enhance this progression, allowing late variants to be generated. In a recent article, Bal de Kier Joffé *et al.* (1986) proposed that a short *in vitro* passage between two *in vivo* transplantations could also induce the selection of more aggressive tumour cells, as the result of an increased synthesis of specific binding proteins. This kind of selection conforms with the evolution of the NT($x$)T lineage. Karyotypic analysis revealed a narrowing of the chromosome content range, suggesting a real selection of cells the best able to survive. Results of the NK lysis sensitivity assay showed that NT($x$)T lineage tumours progressively increased in resistance to NK lymphocytes, but only NT4T cells differed significantly from the parental RMS 9-4/0 cells in all our experiments.

In addition to the selection imposed upon the NT($x$)T lineage, the S($x$)T lineage tumours underwent CZT-induced treatment toxicity and mutagenicity. When successively grafted tumours were kept under constant CZT treatment, progression to severe aggressivity occured. Although the tumour growth rate and the primary tumour sensitivity to CZT were only slightly changed compared to the control NT($x$)T tumour, the spontaneous invasive ability dramatically increased to reach the invasiveness of the RMS 9-4/0 tumour in CZT-treated rats. Furthermore, the liver, a new target organ, was colonized, no metastases being found in this organ in rats bearing tumours induced by the grafting of either RMS 9-4/0, NT($x$)T lineage, or S($x$)T lineage before the 2nd *in vivo* passage. This change in metastatic behaviour occurred concomitantly with a shift in the histological aspect of the tumoral tissue. Fibrous histiocytic neoplasms with predominantly histiocytic differentiation have been described to be highly malignant, and to invade preferentially lung, liver, lymph nodes and mesentery (Greaves & Faccini, 1981). Although the histogenesis of fibrohistiocytic tumours is not yet completely defined, it seems highly unlikely that they are closely related to rhabdomyosarcomas. We ascribe this striking change of S($x$)T lineage behaviour to a treatment-induced genetic alteration. This is supported by the fact that alkylating agents demonstrate mutagenic properties (Bradley *et al.*, 1980; Franza *et al.*, 1980).

The modified histology of S($x$)T lineage tumours correlates with several of our experimental findings. Previous studies (Hart, 1982; Nicolson & Custead, 1982; Tarin & Price, 1981; Tarin *et al.*, 1984) tended to show that it is impossible to adapt metastatic cells to new target organs, but the capacity to disseminate to the liver, as acquired by late S($x$)T passages, is inherent to fibrohistiocytic tumours. Sensitivity to NK cell lysis may be related to the tumour cell stage of differentiation (Werkmeister *et al.*, 1982). Thus, whereas we might have expected an increased resistance of S($x$)T lineage tumours to NK lysis, S2T was significantly more sensitive than RMS 9-4/0 parental cells. Metastatic ability may well increase together with susceptibility to NK lysis, if the number of circulating tumour cells becomes sufficiently high to overcome NK function. Although not significant, a weak selection of more resistant cells appeared to occur over subsequent passages. The narrowing of the chromosome content range of the S($x$)T lineage compared with the NT($x$)T lineage cannot be ascribed to selection by CZT, as we noted that the NT4T tumour was actually as resistant to CZT toxicity as S4T. It is more likely that a new fibrohistiocytic population emerged and became predominant with *in vivo* passages.

However, the histologic divergence of the S($x$)T lineage could explain neither the enhancement of metastatic capacity of RMS 9-4/0 tumours in rats treated with CZT, nor why this effect was not observed when the S4T tumour was treated by CZT. Previous results (Pauwels *et al.*, 1985, 1986) suggest that alkylating agents might act as inducers of proliferation of RMS 9-4/0 and 9-4/0-derived tumour cells. Quiescent disseminated cells may, therefore, have given rise to metastases when treated. This characteristic induced by CZT could have become permanent in S4T cells, thus masking any enhancement of their metastatic behaviour after treatment.

Metastases are known to frequently resist therapy, but only a few reports have described an enhancement of tumour dissemination subsequent to chemotherapy. Various authors (Van Putten *et al.*, 1975; Steel & Adams, 1977) showed that cyclophosphamide pre-treatment could increase, by more than 1000-fold, the number of lung colonies in a mouse mammary tumour model. This effect was related to drug cytotoxicity against host tissue. Lazo *et al.* (1978) found an enhancement of lung colonies after *in vitro* treatment of B16 melanoma cells with ICRF-159, an agent known to reduce *in vivo* metastatic formation, and concluded that the drug had a specific effect on the tumour cells. More recently, McMillan *et al.* (1986) reported a greater than 10-fold increase in the lung colonizing abilities of B16 melanoma sublines treated *in vitro* with hydroxyurea (HU) and allowed to recover 24 h before injection. They related this effect to the mutagenic properties of HU which lead to gene amplification. In all of these experiments, i.v. injection of tumour cells was chosen as the metastatic model. This procedure reproduces the final steps of the metastatic process, that seem to be implicated in the therapy-induced enhancement of tumour dissemination. Our model of spontaneous metastasis includes the necessity for the tumour cells to escape from the primary tumour and enter the vascular system. This could for example involve the motility and the degradative abilities of the tumour cells, as evidenced by our studies of S4T cells *in vitro* (unpublished results). Finally, our work highlights the danger of *in vivo* treatment of well-established tumours with potentially mutagenic chemotherapeutic agents, a problem to which Kerbel and Davies have drawn cancerologists' attention since 1982.

The authors wish to express their appreciation to Brigitte Rosa for the high quality of her histological studies, and to Neil Lynch for his help in improving the English of this manuscript, the unknown referees for their fruitful advice, and Patricia Blanchain for secretarial assistance.

### References

BAL DE KIER JOFFE, E., PURICELLI, L. & DE LUSTIG, E.S. (1986). Modified adhesion behaviour after *in vitro* passage of two related murine mammary adenocarcinomas with different metastasizing ability. *Invasion Metast.*, **6**, 302.

BRADLEY, M.O., SHARKEY, N.A., KOHN, K.W. & LAYARD, M.W. (1980). Mutagenicity and cytotoxicity of various nitrosoureas in V-79 chinese hamster cells. *Cancer Res.*, **40**, 2719.

CIFONE, M.A. & FIDLER, I.J. (1981). Increasing metastatic potential is associated with increasing genetic instability of clones isolated from murine neoplasms. *Proc. Natl Acad. Sci. USA*, **78**, 6949.

FRANZA, B.R., OESCHGER, N.S., OESCHGER, M.P. & SCHEIN, P.S. (1980). Mutagenic activity of nitrosourea antitumour agents. *J. Natl Cancer Inst.*, **65**, 149.

GOLDIE, J.H. & COLDMAN, A.J. (1984). The genetic origin of drug resistance in neoplasms: Implications for systemic therapy. *Cancer Res.*, **44**, 3643.

GREAVES, P. & FACCINI, J.M. (1981). Spontaneous fibrous histiocytic neoplasms in rats. *Br. J. Cancer*, **43**, 402.

HART, I.R. (1982). Seed and soil revisited. Mechanism of site specific metastasis. *Cancer Metast. Rev.*, **1**, 5.

KERBEL, R.S. & DAVIES, A.J.S. (1982). Facilitation of tumour progression by cancer therapy. *Lancet*, **ii**, 1977.

LAZO, J.S., INGBER, D.E. & SARTORELLI, A.C. (1978). Enhancement of experimental lung metastases by cultured B16 melanoma cells treated with (±)-1,2-Bis (3,5-dioxopiperazin-1-yl) propane (ICRF-159). *Cancer Res.*, **38**, 2263.

McMILLAN, T.J., RAO, J. & HART, I.R. (1986). Enhancement of experimental metastasis by pretreatment of tumour cells with hydroxyurea. *Int. J. Cancer*, **38**, 61.

NICOLSON, G.L. & CUSTEAD, S.E. (1982). Tumour metastasis is not due to adaptation of cells to a new organ environment. *Science*, **215**, 176.

NOWELL, P.C. (1976). The clonal evolution of tumor cell populations. *Science*, **194**, 23.

PAUWELS, C., REBISCHUNG, J.L., JASMIN, C. & POUPON, M.F. (1985). Enhanced cloning efficiency of murine rhabdomyosarcoma cells after chlorozotocin treatment: Relationship with enhanced lung metastasis. *J. Natl Cancer Inst.*, **74**, 817.

PAUWELS, C., POUPON, M.F., BREILLOUT, F. & JASMIN, C. (1986). Relation entre capacité métastatique et chimiorésistance des cellules souches clonogènes dans un sarcome de rat. In *Neo Adjuvant Chemotherapy*, Jacquillat, C. *et al.* (eds) p. 61. John Libbey: London.

POUPON, M.F., PAUWELS, C., JASMIN, C., ANTOINE, E., LASCAUX, V. & ROSA, B. (1984). Amplified pulmonary metastases of a rat rhabdomyosarcoma in response to nitrosourea treatment. *Cancer Treat. Rep.*, **68**, 749.

STEEL, G.G. & ADAMS, K. (1977). Enhancement by cytotoxic agents of artificial pulmonary metastasis. *Br. J. Cancer*, **36**, 653.

STEPHENS, T.C., ADAMS, K. & PEACOCK, J.H. (1986). Emergence of nitrosourea resistant sublines of Lewis lung tumours following MeCCNU treatment *in vivo*. *Br. J. Cancer*, **53**, 237.

SWEENEY, F.L., POT-DEPRUN, J., POUPON, M.F. & CHOUROULINKOV, I. (1982). Heterogeneity of the growth and metastatic behavior of cloned cell lines derived from a primary rhabdomyosarcoma. *Cancer Res.*, **42**, 3776.

TALMADGE, J.E. & FIDLER, I.J. (1982). Enhanced metastatic potential of tumor cells harvested from spontaneous metastases of heterogeneous murine tumors. *J. Natl Cancer Inst.*, **69**, 975.

TARIN, D. & PRICE, J.E. (1981). Influence of microenvironment and vascular anatomy on 'metastatic' colonization potential of mammary tumors. *Cancer Res.*, **41**, 3604.

TARIN, D., PRICE, J.E., KETTLEWELL, M.G.W., SOUTER, R.G., VASS, A.C.R. & CROSSLEY, B. (1984). Mechanisms of human tumor metastasis studied in patients with peritoneovenous shunts. *Cancer Res.*, **44**, 3584.

VAAGE, J. (1980). Inherent changes in the *in vivo* growth characteristics of C3H/He mammary carcinomas. *Cancer Res.*, **40**, 3495.

VAN PUTTEN, L.M., KRAM, L.K.J., VAN DIERENDONCK, H.H.C., SMINK, T. & FUZY, M. (1975). Enhancement by drugs of metastatic lung nodule formation after i.v. tumour cell injection. *Int. J. Cancer*, **15**, 588.

WERKMEISTER, J., HELFAND, S.L., HALIOTIS, T., RUBIN, P., PROSS, H. & RODER, J. (1982). Tumour cell differentiation modulates susceptibility to natural killer cells. *Cell. Immunol.*, **69**, 122.